# Supplementary material for: Mechanisms governing the pioneering and redistribution capabilities of the non-classical pioneer PU.1
Source: Nat Commun. 2020 Jan 21;11:402. doi: 10.1038/s41467-019-13960-2 (PMC6972792; doi:10.1038/s41467-019-13960-2)
Supplement: Supplementary file 7 — Source data [file 41467_2019_13960_MOESM7_ESM.zip › Source_Data/FigureS3/FigureS3H_MotifScanOutput/homerResults/motif10.similar.html]

motif10

## Information for motif10

A
G
C
T
G
A
T
C
C
T
G
A
A
T
G
C
A
C
G
T
A
C
G
T
A
C
G
T
A
G
T
C
C
G
T
A
A
G
T
C
C
G
A
T
A
C
G
T
A
G
C
T
A
G
T
C
  
Reverse Opposite:  

T
C
A
G
C
T
G
A
C
G
T
A
C
G
T
A
T
C
A
G
C
G
A
T
C
T
A
G
C
G
T
A
C
G
T
A
C
G
T
A
A
T
C
G
A
G
C
T
C
T
A
G
T
C
G
A
  

|  |  |
| --- | --- |
| p-value: | 1e-77 |
| log p-value: | -1.785e+02 |
| Information Content per bp: | 1.761 |
| Number of Target Sequences with motif | 174.0 |
| Percentage of Target Sequences with motif | 4.50% |
| Number of Background Sequences with motif | 254.5 |
| Percentage of Background Sequences with motif | 0.57% |
| Average Position of motif in Targets | 193.4 +/- 189.4bp |
| Average Position of motif in Background | 210.1 +/- 131.1bp |
| Strand Bias (log2 ratio + to - strand density) | 0.1 |
| Multiplicity (# of sites on avg that occur together) | 1.02 |
| Motif File: | file (matrix) reverse opposite |

### Similar de novo motifs found

|  |  |  |  |  |  |  |  |
| --- | --- | --- | --- | --- | --- | --- | --- |
| Rank | Match Score | Redundant Motif | P-value | log P-value | % of Targets | % of Background | Motif file |
| 1 | 0.946 | C T G A A T G C G C A T A C G T G A C T G A T C C G T A A T G C G C A T G A C T G A C T A G T C G C T A | 1e-73 | -170.252896 | 6.10% | 1.23% | motif file (matrix) |
| 2 | 0.850 | A C G T A C G T A C G T A G T C C T A G A G T C A C G T A C G T A C G T A G T C | 1e-57 | -132.845245 | 4.66% | 0.91% | motif file (matrix) |
| 3 | 0.902 | C G A T T C A G C G T A C T G A C G T A T C A G A C G T C T A G C T G A C G T A C G T A | 1e-53 | -122.791707 | 4.35% | 0.86% | motif file (matrix) |
| 4 | 0.821 | A C G T A C G T A C G T A G T C C T G A A G T C A C G T A C G T | 1e-18 | -42.517218 | 5.87% | 2.98% | motif file (matrix) |
